# Roles of Sodium-Glucose Cotransporter 2 of Mesangial Cells in Diabetic Kidney Disease

**DOI:** 10.1210/jendso/bvab083

**Published:** 2021-05-07

**Authors:** Masanori Wakisaka, Kuniyuki Nakamura, Toshiaki Nakano, Takanari Kitazono

**Affiliations:** 1 Wakisaka Internal Medicine, Fukuoka, 8140013, Japan; 2 Department of Medicine and Clinical Science, Graduate School of Medical Sciences, Kyushu University, Fukuoka, 8128582, Japan

**Keywords:** sodium-glucose cotransporter 2, mesangial cells, tubuloglomerular feedback, chronic kidney disease, diabetic nephropathy

## Abstract

We have been studying the presence of sodium-glucose cotransporter 2 (SGLT2) in mesangial cells and pericytes since 1992. Recent large placebo-controlled studies of SGLT2 inhibitors in patients with type 2 diabetes mellitus have reported desirable effects of the inhibitors on the diabetic kidney and the diabetic heart. Most studies have indicated that these effects of SGLT2 inhibitors could be mediated by the tubuloglomerular feedback system. However, a recent study about urine sodium excretion in the presence of an SGLT2 inhibitor did not show any increases in urine sodium excretion. A very small dose of an SGLT2 inhibitor did not inhibit SGLT2 at the S1 segment of proximal tubules. Moreover, SGLT2 inhibition protects against progression in chronic kidney disease with and without type 2 diabetes. In these circumstances, the tubuloglomerular feedback hypothesis involves several theoretical concerns that must be clarified. The presence of SGLT2 in mesangial cells seems to be very important for diabetic nephropathy. We now propose a novel mechanism by which the desirable effects of SGLT2 inhibitors on diabetic nephropathy are derived from the direct effect on SGLT2 expressed in mesangial cells.

Recent large placebo-controlled trials of sodium-glucose cotransporter 2 (SGLT2) inhibitors (empagliflozin, canagliflozin, and dapagliflozin) in type 2 diabetes mellitus (T2DM) patients observed for approximately 3 to 4 years and other prospective studies of SGLT2 inhibitors in T2DM patients revealed desirable effects of SGLT2 inhibitors [1-9]. However, in those desirable effects, significant and prominent results were restricted to the decreased risks of hospitalization for heart failure, progression of albuminuria, and decrease in glomerular filtration ratio (GFR) [1-5, 11]. Among these effects of SGLT2 inhibitors, the desirable effects of SGLT2 inhibitors on the decrease in albuminuria were reported to be independent of glycemic control [12]. On the other hand, the United Kingdom Prospective Diabetes Study (UKPDS 33) in T2DM patients revealed that attenuation of glycemic control by insulins or sulfonylureas could decrease the risk of progression of microangiopathy during approximately 10 years of observation [13]. A 10-year follow-up of intensive glycemic control in T2DM from the UKPDS revealed a significant 37% decrease in the hazard ratio in microvascular endpoints and a 16% decrease in the hazard ratio in heart failure (HF) during the mean 10.4 years of observation [14]. However, a meta-analysis of strict glycemic control (ADVANCE, UKPDS, ACCORD, and VADT) [15] and studies of dipeptidylpeptidase 4 inhibitors (SAVOR-TIMI 53, EXAMINE, and TECOS) [16-19] did not reveal a beneficial effect on cardiovascular outcome during less than 5 years of observation. In the comparison between these UKPDS findings during 10 years of observation and the constant findings of SGLT2 inhibitors during 2 to 4 years of observations, however, SGLT2 inhibitors seem to have specific functions for the prevention of diabetic nephropathy (DN) and HF in T2DM patients independent of glycemic control [6, 10, 11]. In this review, we would like to focus on the mechanisms of the desirable effects of SGLT2 inhibitors on DN.

## Mesangial Cells

**Figure 1.**
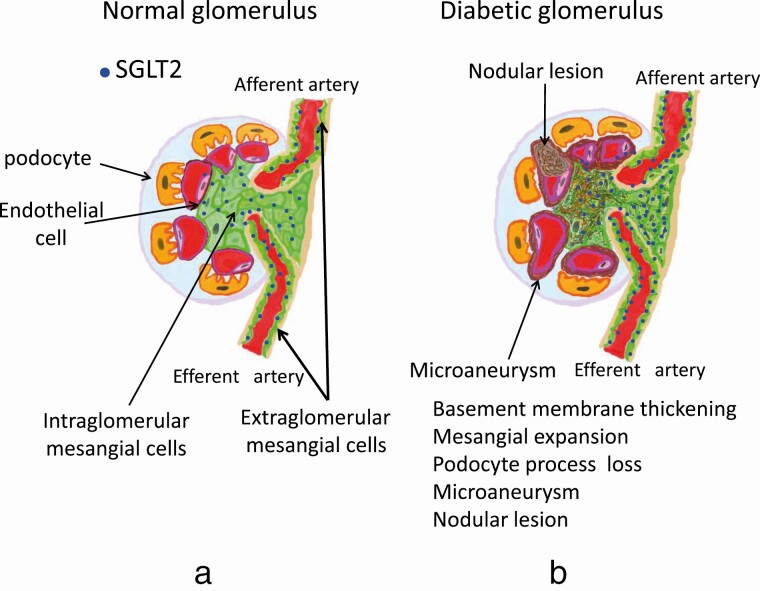
Scheme of diabetic glomerulopathy. (A) Normal glomerulus. Glomeruli consist of three types of cells. Extra- and intraglomerular mesangial cells, glomerular endothelial cells, and podocytes. SGLT2 (●    ) is expressed in mesangial cells. (B) Pathological changes in diabetic glomerulopathy. In early diabetic glomerulopathy, basement membrane thickening and fibrosis occur following mesangial expansion, microaneurysm and nodular lesion formation. SGLT2, sodium-glucose cotransporter 2.

Mesangial cells play important roles in the regulation of glomerular and intraglomerular circulation and the maintenance of glomeruli, such as the protection of glomerular endothelial cells and leakage of substances from serum and fluid from microvessels [20, 21]. Mesangial cells are divided into 2 types of cells by their positions in the glomerulus. One type is the extraglomerular mesangial cells; these cells are present around afferent and efferent arteries of glomeruli. Mesangial cells around afferent arteries maintain constant blood flow in afferent arteries of glomeruli by autocrine adenosine secretion via the tubuloglomerular feedback system [22, 23], and other extraglomerular mesangial cells around efferent arteries of glomeruli react to angiotensin II derived from the renin angiotensin system (RAS) [24]. Other mesangial cells are intraglomerular mesangial cells, and these cells protect intraglomerular endothelial cells, regulate glomerular circulation, maintain glomerular structures, and protect against the leakage of serum substances, such as albumin, and fluid from microvessels in the glomerulus (Fig. 1A). In DN, an increased GFR and basement membrane thickening are reported to occur following glomerular expansion and decreased GFR [25, 26]. In the early stage of DN, the increase in the GFR is explained by the glomerular hemodynamic hypothesis [27] or tubuloglomerular feedback system [28], of which mechanisms are based on the balance between glomerular afferent and efferent arteriolar tone [29]. In the diabetic state, rat mesangial cells are reported to lose their contractile response to contractive substances in vitro, such as angiotensin II [30], followed by mesangial cell swellings, which are thought to induce glomerular hyperfiltration and microaneurysms, as seen in diabetic retinopathy (DR), in the glomerulus [31-34]. An in vitro study indicates that rat mesangial cells are also implicated in hyperfiltration and glomeruli, which stem from cellular contractile dysfunction [31, 35]. Our in vitro study using rat mesangial cells demonstrated that an increase in extracellular glucose concentration induced mesangial dysfunctions, such as loss of contractile response to contractive substances, overproduction of extracellular matrix and apoptosis. These changes are well-known in diabetic glomerulopathy [36], in which accumulation of extracellular matrix induces basement membrane thickening and fibrosis, following mesangial expansion, microaneurysm, and nodular lesion formation, resulting in diabetic-specific structural changes (Fig. 1B). Interestingly, phlorizin, an SGLT inhibitor, was reported to normalize the high glucose-induced loss of the contractile response of rat mesangial cells and cellular swellings in vitro [36]. From these observations, SGLT2 might be present in mesangial cells, and the desirable effects of SGLT2 inhibitors in DN independent of glycemic control might be derived from the direct action of SGLT2 inhibitors on SGLT2 in mesangial cells.

## Tubuloglomerular Feedback

**Figure 2.**
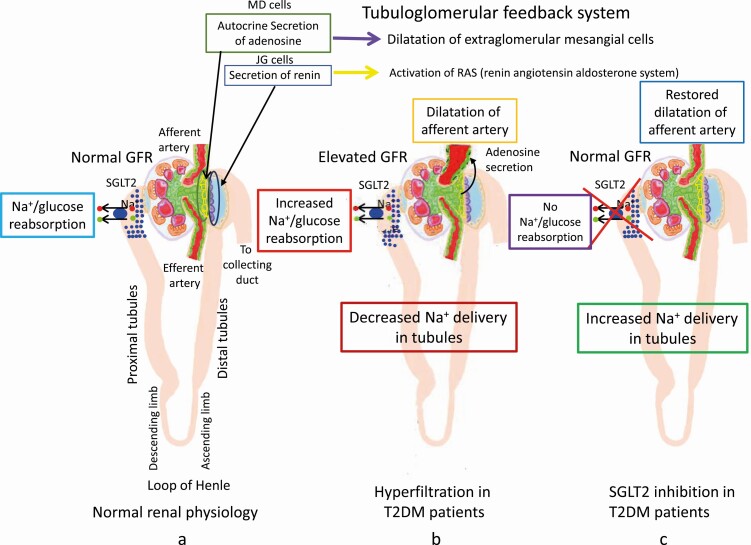
Tubuloglomerular feedback hypothesis for the underlying mechanism of pleiotropic effects by SGLT2 inhibitors. JG, juxtaglomerular cells; MD, macular densa cells. (A) In nondiabetic subjects, glomerular filtration ratio (GFR) is maintained normal through tubuloglomerular feedback system. (B) In T2DM patients, increased reabsorptions of glucose and Na^+^ by SGLT2 in proximal tubules cause a decrease of urinal Na^+^ excretion at the same time, which stimulates an autocrine secretion of adenosine through tubuloglomerular feedback system. The secreted adenosine dilates afferent arteriole of glomeruli and induce elevated GFR. (C) In T2DM patients treated with SGLT2 inhibitors, reabsorptions of glucose and Na^+^ by SGLT2 in proximal tubules are inhibited completely and then the urinal Na^+^ excretion is increased again, which restores the dilatation of the afferent arteriole of glomeruli and elevated GFR through tubuloglomerular feedback when the presence of SGLT1 in proximal tubules is not taken account. SGLT1, sodium-glucose cotransporter 1; SGLT2, sodium-glucose cotransporter 2; T2DM, type 2 diabetes mellitus.

The juxtaglomerular (JG) apparatus in the nephron consists of 3 types of cells, macula densa (MD), JG, and extraglomerular mesangial cells, to regulate renal blood flow and the GFR. Recent large placebo-controlled studies of SGLT2 inhibitors in patients with T2DM have reported desirable effects of these inhibitors on DN [1-5, 10, 11]. Most studies proposed that these effects of SGLT2 inhibitors could be mediated by tubuloglomerular feedback [1-5, 10, 11]. Their explanations are as follows [37] (Fig. 2). In the control subjects, GFR is maintained normal through tubuloglomerular feedback system (Fig. 2A), in which MD cells sense Na^+^ and Cl^-^ concentrations in distal tubules and control autocrine secretion of adenosine, which dilates afferent arterioles of the glomerulus via relaxation of extraglomerular mesangial cells [38]. JG cells secrete renin in response to decreased renal perfusion pressure or Na^+^ and Cl^-^ concentrations. In states with diabetes mellitus, increased glucose and Na^+^ reabsorption by SGLT2 occurs at the S1 segment of the proximal tubules, and the consequential decrease in intratubular Na^+^ concentrations at MD cells set tubuloglomerular feedback in motion and leads to afferent arteriole dilatation (Fig. 2B). This increased reabsorption of Na^+^ would promote Na^+^ overload in the body and hyperfiltration in the glomeruli, resulting in HF and DN, respectively. SGLT2 inhibitors, by inhibiting Na^+^ and glucose reabsorption at the S1 segment in proximal tubules, revert the pathologic process to normal GFR by increasing the tubular Na^+^ concentration (Fig. 2C). However, there was an opposite suggestion regarding the renal protective effect of empagliflozin by tubuloglomerular feedback [39]. The tubuloglomerular feedback hypothesis raises the following serious concerns.

First, the authors of the tubuloglomerular feedback system hypothesis paid no attention to other Na^+^ reabsorption mechanisms operating in the tubular system. Na^+^ is reabsorbed by many mechanisms, such as SGLT1, Na^+^ channels, Na^+^-phosphate cotransporters, and Na^+^-H^+^ exchangers. If the function of SGLT2 is blocked, other Na^+^-reabsorbing mechanisms should be set in motion to maintain homeostasis, especially SGLT1 in the S3 segment of proximal tubules according to the report from Ab-Ghami et al. [40].

**Figure 3.**
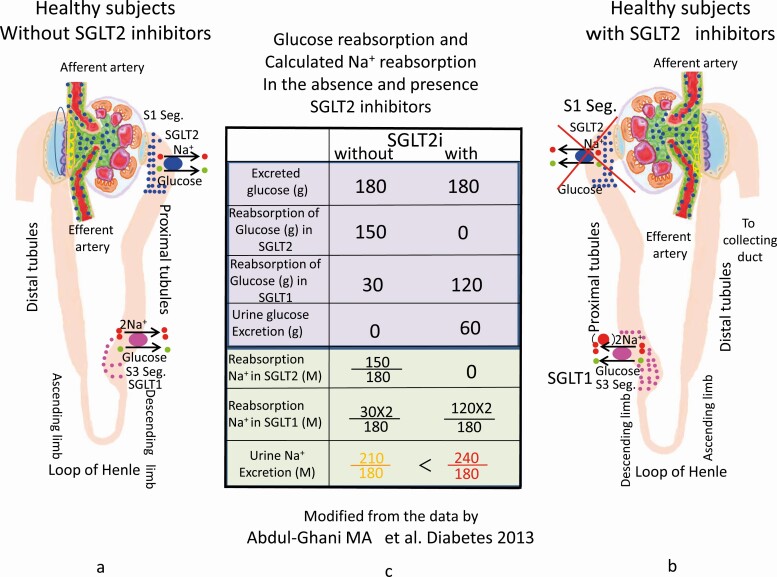
Glucose reabsorption and calculated Na^+^ reabsorption and excretion in proximal tubules in the absence and presence SGLT2 inhibitors. SGLT1 (●     ) and SGLT2 (●    ) in proximal tubules. The reabsorption data of glucose by SGLT2 and SGLT1 in this figure are derived from Abdul-Ghani MA et al. [40], which were permitted by American Diabetes Association to use for our Figure 3. (A) Glucose and Na^+^ reabsorption in proximal tubules by SGLT1 and SGLT2 in the absence of SGLT2 inhibitors. The stoichiometry of glucose and Na^+^ is 1:2 in SGLT1 and 1:1 in SGLT2, respectively. (B) Glucose reabsorption in proximal tubules in the presence of an SGLT2 inhibitor. When SGLT2 is inhibited in the S1 segment, upregulation of SGLT1 results in increased glucose and Na^+^ reabsorption in the S3 segment. (C) Calculated Na^+^ and glucose reabsorption. Data are based on the report from Abdul-Ghani MA et al. *Diabetes* 2013. From these calculations, increased glucose reabsorption by SGLT1, which means the increase of Na^+^ reabsorption, results in the decrease of urinal Na^+^ excretion in the presence of SGLT2 inhibitors. Finally, total excretion of Na^+^ in the presence of SGLT2 inhibitors exceeds that in the absence of SGLT2 inhibitors. SGLT1, sodium-glucose cotransporter 1; SGLT2, sodium-glucose cotransporter 2.

Here, we theoretically assessed Na^+^ reabsorption from glucose dynamics in the proximal tubule. In a healthy man, 180 g/day glucose is filtered into the proximal tubule, and SGLT2 reabsorbs 150 g of glucose in the S1 segment, which means that SGLT1 in the S3 segment reabsorbs 30 g of glucose to make daily urinary glucose excretion 0 g [40] (Fig. 3A). In the presence of an SGLT2 inhibitor, SGLT1 would be upregulated to compensate for disrupted glucose transport, since both SGLTs are regulated by protein kinase C (PKC) in almost the same manner [41]. SGLT1 should take up 120 g of glucose because 60 g of glucose appears in urine in a healthy man taking an SGLT2 inhibitor [40] (Fig. 3-b). The coupling ratio of glucose to Na^+^ is 1:1 in SGLT2 and 1:2 in SGLT1. As a consequence, Na^+^ reabsorption is estimated as 150/180 (by SGLT2) + 2 × 30/180 (by SGLT1) = 1.16 moles in the absence of an SGLT2 inhibitor. In a similar fashion, 0 + 2 × 120/180 = 1.33 moles of Na^+^ is reabsorbed in the presence of an SGLT2 inhibitor (Fig. 3C). Thus, SGLT2 inhibitors increase the total proximal tubular reabsorption of Na^+^, which means the decrease tubular Na^+^ excretion (Fig. 3C). In support of this, decreased urinal Na^+^ excretion with an SGLT2 inhibitor was reported in diabetic rats [42].

Second, the Na^+^ fraction reabsorbed by SGLT2 would be too small to influence tubuloglomerular feedback. In a person with 180 L/day GFR and 140 mEq/L serum Na^+^, approximately 25.2 (= 180 × 0.14) moles of Na^+^ are reabsorbed because more than 99% of filtered Na^+^ is reabsorbed in the tubular system. When 90 g/day (0.5 moles) glucose is excreted in urine from a patient using an SGLT2 inhibitor, the same amount of Na^+^ (0.5 moles) is left unabsorbed together with glucose, which corresponds to only 2% of total filtered Na^+^ (100 × 0.5/25.2). Such a small change in Na^+^ concentration is unlikely to affect glomerular hemodynamics to a considerable degree. Again, the small amount of Na^+^ left unabsorbed would be further decreased by other Na^+^ reabsorption mechanisms.

Furthermore, another argument is the change in tubular Cl^-^ concentrations, which is the critical determinant in the regulation of tubuloglomerular feedback. Because SGLT2 inhibitors increase Na^+^ reabsorption in proximal tubules as mentioned previously, tubular Cl^-^ shifts as well as Na^+^ and consequently tubular Cl^-^ concentrations will decrease, which sharply contrasts with the proposed tubuloglomerular feedback theory. In addition, it is unknown whether autocrine adenosine secretion in tubuloglomerular feedback dilates afferent arteries that consist of damaged mesangial cells in the diabetic state, as we mentioned [36]. A recent study about combination therapy with SGLT2 inhibitors in T2DM patients taking loop diuretics because of HF revealed no increase in urinal sodium excretion [43], which means urine Na^+^ uresis is not increased in the treatment with SGLT2 inhibitors.

Finally, the DAPA-CKD trial and DAPA-HF trial revealed reductions in the progression risk of chronic kidney disease (CKD) and HF with reduced ejection fraction in both patients with and without diabetes mellitus [44, 45]. In nondiabetic subjects, an increased GFR is unlikely to occur (i.e., tubuloglomerular feedback theory may not apply). In the DAPA-CKD study, the authors speculated the presence of hyperfiltration by decreases in nephron CKD [44]. Because all nondiabetic patients with CKD or HF do not have severely reduced estimated GFR based on the data of these trials, decreases in the numbers of nephrons in most subjects seem to be absent, which does not mean the presence of hyperfiltration in those patients. Therefore, risk reductions by dapagliflozin, an SGLT2 inhibitor, in the progression of CKD and HF among most of the subjects without T2DM are also independent of hyperfiltration and tubuloglomerular feedback. Concerning the effects of SGLT2 inhibitors on HF, we refer to another review [46].

Thus, the tubuloglomerular feedback theory has been widely believed to attenuate the diabetic kidney and heart by SGLT2 inhibitors; however, SGLT2 inhibitors may not induce Na^+^ uresis and may not increase the Na^+^ concentration in tubules in the kidney than previously expected. The tubuloglomerular feedback theory seems to be independent of the reasons for the desirable effects of SGLT2 on DN and HF in T2DM patients.

## Renin Angiotensin System

In the RAS, the blood flow in the glomeruli is decreased, and JG cells secrete renin, which produces angiotensin II by angiotensin-converting enzyme, contracting glomerular efferent arterioles. Thus, the RAS system induces intraglomerular hypertension in DN [47]. Inhibition of the RAS by angiotensin-converting enzyme inhibitors (ACEIs) or angiotensin receptor blockers were reported to reduce the incidence of albuminuria in T2DM patients [48, 49] and to decrease the risk of DN and DR in diabetics [49, 50]. These observations might suggest desirable effects particularly in the early stages of DN and DR. However, in the condition of decreased contractile response because of SGLT2 under high glucose [36], whether contractile agents, such as angiotensin II, could affect the mesangial cell contractile response is unknown. Interestingly, ACEIs were reported to decrease proximal tubular SGLT2 protein levels compared with those in control diabetic rats in vitro [51]. Although the concentration of captopril (an ACEI) seems to be high (10^-4^ M), it was reported to inhibit SGLT2 in bovine retinal pericytes in vitro [52]. From these data, RAS inhibitors might act as weak SGLT2 inhibitors and might attenuate DN.

## SGLT2 in Mesangial Cells

Extracellular sodium-dependent and phlorizin (a nonspecific SGLT inhibitor)-sensitive glucose uptake by rat mesangial cells in vitro was reported in 1995 [53]. The expression of SGLT2 in rat mesangial cells determined by western blot analysis in vitro was reported [36]. SGLT2 expression in mesangial cells from dB/dB mice increased approximately 5-fold under high-glucose conditions in vitro [54]. SGLT2 expression was reported to be increased by PKC [55]. Because PKC was reported to increase expressions of SGLT2 [41, 55], the increased expression of SGLT2 in dB/dB mice under a high-glucose condition and a diabetic state was derived from the increase in PKC in those circumstances. The increased glucose uptake as well as Na^+^ through SGLT2 by mesangial cells would raise intracellular sorbitol levels through the polyol pathway and activate PKC through the diacyl-glycerol PKC pathway, leading to inhibition of Na^+^/K^+^-ATPases [56]. As a result, swelling, dysfunction, and loss of the cells occur [36]. These changes cause the formation of microaneurysms, which are characteristic changes in diabetic microangiopathies, as often seen in DR [57, 58]. Moreover, microaneurysms were observed in the human diabetic heart [59] and were also observed in DN [32-36]. High-glucose conditions induce mesangial cell swelling and loss of the contractile response, and these cellular dysfunctions are a cause of hyperfiltration in the early stages of DN [36]. These dysfunctions of mesangial cells under high-glucose conditions were restored by phlorizin [36, 59]. In a recent report, a very low dose of canagliflozin (an SGLT2 inhibitor) attenuated glucose consumption by mesangial cells of db/db mice under high-glucose conditions [54]. Moreover, the low dose of the SGLT2 inhibitor normalized TGF-β1 and fibronectin mRNA levels in the mesangial cells without lowering serum glucose and fructosamine levels. These results suggest that even a very low dose of canagliflozin directly normalized glucose uptake through SGLT2 in mesangial cells following normalization of intracellular glucose levels and TGF-β1 and fibronectin mRNA levels. These beneficial effects of canagliflozin normalized albuminuria and pathological changes in DN without changing glucose levels (the same high-glucose levels as those in db/db mice). This very low dose of canagliflozin is never excreted in urine and could not inhibit SGLT2 at the S1 segment of proximal tubules in db/db mice [54] because the excretion of canagliflozin in urine was <1% of the administered dose. These observations seem to indicate that renoprotection by SGLT2 inhibitors can be explained by their direct action on SGLT2 in mesangial cells in DN, which means that the effects of SGLT2 inhibitors on DN are independent of tubuloglomerular feedback.

## SGLT2 in Other Cells

Expression of SGLT2 other than in proximal tubular cells and mesangial cells was also reported in bovine retinal pericytes in vitro [52, 60-62]. The origin of both mesangial cell and pericytes is considered to be mesenchymal stem cells. Glomerulus in the kidney consists of mesangial cells, podocytes, and endothelial cells, whereas microvessels in the body consist of pericytes and endothelial cells [63]. From the published data, SGLT2 acts as a physiological glucose sensor, and Na^+^ and glucose enter through SGLT2 depending on the extracellular glucose concentration. Na^+^ taken up through SGLT2 in bovine retinal pericytes in vitro is exchanged by Ca^2+^ via a sodium-calcium exchanger; thus, extracellular glucose concentration-dependent Ca^2+^ entry occurs, which regulates the tone of the pericytes (i.e., extracellular high glucose induces the pericyte contraction and extracellular low glucose induces the pericyte dilatation) [62]. However, this physiological function of SGLT2 in pericytes is abolished by the presence of SGLT2 inhibitors. Under high-glucose conditions, sorbitol and PKC, as mentioned in the paragraph on SGLT2 in mesangial cells of this manuscript, inhibit Na^+^/K^+^ ATPase, and these inhibitions seem to induce cellular swelling and apoptosis of retinal pericytes following microaneurysm in the retina, namely DR. Because capillaries consist of endothelial cells and pericytes and are present in most organs, pericytes are thought to play important roles in peripheral tissues in the human body. Under high-glucose conditions, renal interstitial lesions are always exposed to hypoxia, and blood supplies to these lesions are mainly capillaries. Because capillary consist of endothelial cells and pericyte, in these circumstances, pericytes in the kidney might play also important roles in renal interstitial lesions in diabetes mellitus and CKD.

On the other hand, SGLT2 expression was reported to be absent in bovine retinal endothelial cells in vitro [61]. In glomeruli in the kidney, the glomerulus consists of mesangial cells, endothelial cells, and podocytes. At present, the expression of SGLT2 in podocytes is unknown. Because dysfunctions of podocytes, such as loss of foot processes, are well known to occur in the diabetic state, the presence or absence of SGLT2 in podocytes should be clarified.

## SGLT2 Inhibitors in the Kidney

The structures of the kidney consist of numerous nephrons from the glomerulus to the collecting duct [64]. Regarding DN, the effects of SGLT2 inhibitors on diabetic glomerulopathy are mentioned in the paragraph on mesangial cells in this review; however, interstitial tubular injury was reported to develop before glomerular dysfunction [65, 66]. Moreover, tubule-interstitial fibrosis is also considered as a characteristic pathological change of the early stage of DN [67]. Renal interstitial cells always function as exchangers of various ions or reabsorbs of various ions and substances, such as glucose [41, 68, 69]. Under diabetic conditions, however, these cells are exposed to hypoxia and the activated HIF-1 leads to fibrosis [70-72]. Hypoxia in the renal interstitial lesion under diabetic states was also reported to decrease erythropoietin production by dysfunctions of neural crest cells derived from the fibroblasts surrounding the renal tubules [73]. In T2DM patients, the erythropoietin level showed increase after initiation of treatment with an SGLT2 inhibitor dapagliflozin [74]. Other beneficial effects of SGLT2 inhibitors on renal interstitial lesions were furthermore reported [75-77]. In interstitial fibrosis, loss of pericytes in the capillary of interstitial lesion were suggested to play a crucial role in breakdown of proper capillary functions [78]. These beneficial effects of SGLT2 inhibitors are supposed to be derived from attenuation of microcirculation. Therefore, it may be difficult to explain these effects simply by the tubuloglomerular feedback theory, and pericytes in renal interstitial lesions should be taken into consideration. The expression of SGLT2 in bovine retinal pericytes was reported [46, 60-62], as mentioned for SGLT2 in other cells previously. From our long-time experiments of pericytes, we suggest the presence of SGLT2 in pericytes, and beneficial effects of SGLT2 inhibitors on renal interstitial lesions seem to be derived from direct effects of the inhibitors on SGLT2 in renal interstitial pericytes. Thus, SGLT2 in mesangial cells and pericytes in the kidney seems to be directly affected by SGLT2 inhibitors, which have beneficial effects on the kidney with and without diabetes.

## Other Mechanisms Other Than a Direct Effect of SGLT2 Inhibitors on SGLT2 Expressed in Mesangial Cells

In the early period of SGLT2 inhibitor clinical use, causes of its desirable effects of SGLT2 inhibitors were raised, such as lowering blood glucose, increased whole-body metabolism from glucose to fat oxidation, increased ketone concentration, decreased uric acid concentration, increased plasma glucagon concentration, body weight loss changes in plasma electrolyte concentration, and decreased in blood pressure and [79]. However, these factors, other than the decrease in blood pressure, were reported to be unlikely causes of the preferable effects of SGLT2 inhibitors [79]. Recently, effects of SGLT2 inhibitors on attenuation of oxidative stress in T2DM patients [80], reduction of inflammation in rat in vivo [81], and inhibition of Na^+^/H^+^ exchanger in rat and rabbit in vitro [82, 83] are raised for the explanations for preferable effects of SGLT2 inhibitors. However, a definite mechanism is still unknown.

## Conclusion

In recent large, placebo-controlled trials of SGLT2 inhibitors and other prospective studies concerning the heart and kidney during short observation periods, the prominent desirable effect on HF, albuminuria, and decrease in GFR were clarified. Most studies reported that those desirable effects were independent of glycemic control, and the mechanisms of the effect of SGLT2 inhibitors are controversial. The tubuloglomerular feedback system has been mainly used for the explanation for the desirable effects; however, from this review, the tubuloglomerular feedback system may not be a good explanation for the desirable effects of SGLT2 inhibitors on the heart and kidney in T2DM patients. Here, we propose a novel mechanism underlying the desirable effects of SGLT2 inhibitors. These effects would be most properly explained if we assume direct actions of the inhibitors on SGLT2 in mesangial cells because expressions of SGLT2 in mesangial cells were reported experimentally. Because mesangial cells regulate microcirculation and maintain the physiological structures of microvessels in renal glomeruli, pathological changes in diabetic glomerulopathy and albuminuria were attenuated by the inhibition of SGLT2 in mesangial cells as a result. Moreover, SGLT2 in pericytes might take part in the progression of renal interstitial dysfunctions in diabetic states because SGLT2 is expressed in bovine retinal pericytes, and dysfunction of pericytes in the renal interstitial lesion may be the causes of DN. Additional precise investigations are needed to clarify the expression of SGLT2 in the concerned cells.

## Data Availability

All data analyzed during this review are included in this published article or in the data repositories listed in References.

## Abbreviations

ACEI: angiotensin-converting enzyme inhibitor
CKD: chronic kidney disease
DN: diabetic nephropathy
DR: diabetic retinopathy
JG: juxtaglomerular
GFR: glomerular filtration ratio
HF: heart failure
MD: macula densa
PKC: protein kinase C
RAS: renin angiotensin system
SGLT2: sodium-glucose cotransporter 2
T2DM: type 2 diabetes mellitus
UKPDS: United Kingdom Prospective Diabetes Study.

## Additional Information

***Disclosures:*** M.W., K.N., and T.N. declare no competing interest. T.K. provided consultations or received lecture fees from Chugai Pharmaceutical Co Ltd., and grant support from Takeda Pharmaceutical Co Ltd., Daiichi Sankyo Ltd., Mitsubishi Tanabe Pharma Co, Eisai Co Ltd., Astellas Pharma Inc, Chugai Pharmaceutical Co Ltd., and MSD KK.
